# Nicotine dependence in an isolated population of Kashubians from North Poland: a population survey

**DOI:** 10.1186/s12889-015-1455-5

**Published:** 2015-02-04

**Authors:** Alicja Sieminska, Ewa Jassem, Karolina Kita-Milczarska

**Affiliations:** Department of Allergology and Pneumonology, Medical University of Gdansk, Debinki Str. 7, 80-211 Gdansk, Poland

**Keywords:** Nicotine dependence, Population survey, Demographic, Nicotinic receptor subunit, Polymorphism

## Abstract

**Background:**

Tobacco use is a complex, multistage behaviour. The particular stages of this behaviour, including nicotine dependence (ND), are influenced by both genetics and the environment. Surveys on factors influencing tobacco use and ND, conducted in ethnically homogenous populations, can provide results less influenced by genetic and cultural heterogeneity. We aimed to assess ND in a sample of current smokers, derived from the geographically and culturally isolated population of Kashubians from North Poland, and evaluate its potential association with age, sex, and self-reported comorbidities. In addition, we attempted to replicate - for the first time in this population - previous findings on the association between ND and several variants within the *CHRNA5A3*-*A5*-*B4* nicotine receptor subunit gene cluster.

**Methods:**

The study sample consisted of 969 unrelated subjects who were all current smokers. ND was evaluated using four measures: the Fagerstrom Test for Nicotine Dependence (FTND), the Heavy Smoking Index (HSI), the number of cigarettes per day (CPD) and the time to first cigarette after waking (TTF). All subjects underwent genotyping for *CHRNA5* rs16969968, *CHRNA3* rs578776, and *CHRNB4* rs12914008 variants. Multivariate regression analysis was used for the assessment of the studied correlations. A significance level of 0.05 with the Bonferroni correction for multiple testing was set for a type 1 error in the analyses.

**Results:**

The mean CPD, FTND and HSI scores in the study sample were 17.3 ± 7.7, 3.9 ± 2.3 and 2.6 ± 1.5, respectively. No association between ND defined by FTND, HSI or TTF and age was found. In turn, heavy smoking was significantly associated with older age (odds ratio (OR) = 1.72, 95% confidence interval (CI): 1.14-2.59, p = 0.009), and men were more likely than women to be heavy smokers (OR = 1.70, 95% CI: 1.09-2.65, p = 0.018). Chronic comorbidity did not significantly influence ND. An analysis of association of studied polymorphisms with ND showed a borderline association of rs16969968 with CPD (OR = 1.63, 95% CI: 1.09–2.45, p = 0.017).

**Conclusion:**

Our study showed a low to moderate level of ND in the Kashubians, influenced by age, sex, as well as the *CHRNA5* rs16969968 variant.

## Background

Tobacco use is one of the most dangerous habits, and as it is widespread across the world, killed almost 6 million people in 2011 [1]. Unfortunately, smoking rates are increasing in some developing countries, and still remain high in many developed countries despite heightened public awareness of the health risks and public health efforts that have been made to reduce cigarette consumption. According to the estimates of the World Health Organization, if current trends continue, by the year 2030, eight million people will die annually from tobacco smoking, making this habit the single largest cause of mortality worldwide [1]. However, the premature deaths and significant morbidity associated with smoking can be entirely preventable; therefore, more measures must be undertaken worldwide to stop the tobacco epidemic. Studies identifying the factors influencing tobacco dependence and that are the leading predictors of smoking continuation will likely play a significant role in achieving this goal [2].

Tobacco use is a complex, multistage behaviour that includes initiation, experimentation, regular use, dependence, cessation, and relapse [3,4]. This behaviour is influenced by both genetics and the environment [5]. Classical genetic twin studies, allelic association studies on candidate genes, and more recently, genome wide association studies (GWAS), have yielded strong evidence on the substantial role of genetics in phenotypes displayed at every stage of this behaviour, including nicotine dependence (ND) [6-13]. In the last decade, the genetic region that may be involved in the pathogenesis of ND was identified to be present within chromosome 15 [12,13]. This genome region comprises a cluster of genes encoding the CHRNA5, CHRNA3, and CHRNB4 subunits of the neuronal nicotinic acetylcholine receptor (nAChR) [12,13]. nAChRs in the central nervous system largely mediate the effects of nicotine, the primary substance responsible for the addiction that contributes to maintaining tobacco use [14]. After binding to nAChRs, nicotine stimulates dopamine release in the striatum, a region of the brain that is involved in the reward pathway and is crucial to the development of substance dependence [15-17]. Several genome-wide association and candidate genes studies have provided further evidence supporting the relationship of different loci within this gene cluster with various ND phenotypes the number of cigarettes smoked daily (CPD), the Fagerström Test for Nicotine Dependence (FTND) [18] score, and FTND-derived measures such as the Heavy Smoking Index (HSI) [19] and the Time to First Cigarette (TFF) or the age at starting daily smoking [13,20-25].

Most frequently, a significant association of ND with the rs16969968-tagged locus, or single nucleotide polymorphisms (SNPs) that are highly correlated with it (r^2^ ≥ 0.8), was found in numerous independent studies in subjects of Caucasian origin [20-22,26]. Owing to this strong evidence of the contribution of rs16969968 to ND, especially when defined by CPD, this association seems to be stably established [27,28]. In addition, several distinct loci that have low linkage disequilibrium (LD) with rs16969968, including rs578776 and rs12914008, were found to influence nicotine-related phenotypes [12,13,20,22-24,29].

The environment is the second component influencing tobacco use and ND phenotypes. The most important environmental factors include socio-cultural influences, which are specific to different ethnic or geographical populations [30,31]. Therefore, there is a considerable cultural variation in tobacco use worldwide. In addition, the influence of ethnic and cultural factors on tobacco use phenotypes can be modified by genetic factors, which can exert different effects across different populations because SNP allele frequencies and their association with ND can differ between various ethnic or geographic populations and subpopulations owing to variations in the extent of LD in these different groups. The effects of genes that contribute to tobacco use and ND might be enhanced in isolated, genetically homogeneous populations that are characterized by limited allelic diversity.

Kashubians are a relatively small population that inhabits Kashubia, North Poland; a region in Poland’s Pomeranian Province. Currently, the number of indigenous Kashubians living in this region is estimated at nearly 230,000 [32]. Kashubians are considered to be an isolated population, although the genetic structure of this population has not yet been exhaustively studied. The assumed isolation of Kashubians is based mainly on cultural and linguistic reasons, as well as on the geographical location of Kashubia in the outskirts of North Poland. However, a study of the history of Kashubians across centuries suggested that this population might instead be classified as secondarily isolated, i.e., isolated after being detached from a large population, which then slowly expanded with very little recruitment from outside the group. Kashubians are first mentioned in historical records from the middle of the thirteenth century; however, the age of this population is not precisely known. Nevertheless, several lines of evidence in the Kashubian population conform to the criteria of an isolated population: an old settlement, high rates of endogamy with consanguineous marriages between distant relatives, and slow population expansion with negligible immigration, accompanied by the conservation of a strong socio-cultural identity, including distinct dialect and traditional customs.

In addition to genetic and environmental factors, different smoker’s individual factors, including comorbidities, can contribute to tobacco use and ND [5]. Therefore, the aim of our study was to assess ND in a convenience sample of current smokers of Kashubian descent, and to correlate results with the basic demographic variables and concomitant chronic conditions. In addition, we attempted to replicate - for the first time in this population - previous findings on the association between several variants within the *CHRNA5A3*-*A5*-*B4* gene cluster and ND.

## Methods

### Ethics statement

The institutional research ethics committee at the Medical University of Gdansk approved all study procedures, and all subjects provided written, informed consent prior to participation in the study.

### The study sample

The convenience sample was selected from both the consecutive outpatients attending several health centres and blood donors attending blood donation services in Kashubia. Participants were adult, non-related, current cigarette smokers, who self-identified as being of Kashubian descent. A current smoker was defined as someone who self-reported smoking cigarettes daily or occasionally for at least 1 year at the moment of recruitment to the study. The recruitment of eligible subjects of Kashubian origin was conducted by nurses, who are skilled in questionnaire face-to-face interviews for collecting demographic, genealogical, and smoking habit data. In addition, information on the presence of any chronic diseases, including psychiatric disorders, was obtained from participants by self-reporting. The Kashubian origin of participants was confirmed by being born into a Kashubian family (i.e., both a mother and a father, as well as four grandparents were Kashubian) and by a command of the Kashubian language. In order to exclude first-degree relatives, every consecutive participant was asked to list their living parents, adult siblings, and offspring in the questionnaire. The information of all of these relatives was then collected in a separate database. Upon recruitment of each new subject, this database was consulted to check whether the new potential recruit had already been recorded as a first-degree relative of any other previously recruited subject. If two subjects were found to be first-degree relatives, only the younger subject, preferentially without any self-reported chronic disease, was included in the study.

Initially, 1110 subjects who were current smokers and self-identifying as being of Kashubian descent were recruited into the study. Finally, after excluding 141 (12.7%) subjects who did not fulfill the criteria of definite Kashubian origin or appeared to be first-degree relatives of any other participant, the study population comprised 969 subjects: 748 (78%) subjects who had been recruited in health centres and 221 (23%) blood donors.

### Nicotine dependence measures

ND was assessed by the Fagerström Test for Nicotine Dependence (FTND), the measure that conceptualized nicotine dependence as a syndrome characterized by physiological, cognitive, and behavioral symptoms. The FTND has six items, and therefore, the scores are commonly treated as continuous variables with the dependence status varying in degree.

The Heaviness of Smoking Index (HSI), which is derived, similarly to FTND, from the Fagerström Tolerance Questionnaire [33] was also recorded in each participant. HSI is considered a brief version of the FTQ or FTND, which retains their two main items: the number of cigarettes smoked per day (CPD) and the time to first cigarette after waking (TTF). The values for HSI as the sum of these two categorical measures range from 0 to 6, which correspond to the following for CPD: 0: 0–10 CPD; 1: 11–20 CPD; 2: 21–30 CPD; and 3: 31+ CPD. The HSI scores for TTF are: 0: 61+ min; 1: 31–60 min; 2: 6–30 min; and 3: up to 5 min.

Heavy smokers were defined as those who smoked more than 20 cigarettes daily, and chippers were defined as smokers who smoked occasionally or up to five cigarettes daily [34].

### Sample collection procedures and genotyping

Upon recruitment of each subject, an 8-mL venous blood sample was collected into heparinized tubes. Samples were frozen and stored at −80°C until required for molecular genotypic analyses. Based on previous findings, three SNPs for ND were selected for genotyping: rs16969968 in *CHRNA5*, rs578776 in *CHRNA3*, and rs12914008 in *CHRNB4* [12,13,20-29].

Genomic DNA was extracted from lymphocytes and used as a template for real-time polymerase chain reaction (PCR). DNA isolation was performed with the use of the Micro Blood Gravity kit (A&A Biotechnology), according to the manufacturer protocol. The DNA samples were then diluted 10 times in 10 mM Tris buffer, pH 8.0, and stored at 4°C. PCR amplification was carried out in a total volume of 30 μL by using 5 μL of the diluted DNA and Real Time 2x PCR Mix SYBR A or Real Time 2x PCR Mix EX SYBR A (A&A Biotechnology). PCR was carried out in the iCycler system (Bio-Rad) using SYBR Green as a fluorochrome. Cycling was carried out with an initial denaturation at 95°C for 3 min followed by 40 cycles of 95°C for 15 s and 62–68°C for 1 min. The amplified DNA fragments were 130–350 bp. Melt curves were generated by holding the PCR plate at 95°C for 1 min followed by 55°C for 10 s. The temperature was then increased every 0.5°C to the final temperature of 95°C using step fluorescence acquisition. Melt curve profiles were assessed and analyzed using iCycler software. To validate the method, selected samples were resolved on 2% agarose gel.

### Statistical analyses

The Hardy-Weinberg Equilibrium (HWE) test was performed with Haploview software (version 4.2) [35]. The chi-squared (χ^2^) test with continuity correction whenever appropriate was used for group comparisons of allele and genotype frequencies.

Multivariate logistic regression analysis was applied with the use of STATISTICA 10.0 software (StaSoft Inc.; USA) to estimate correlations between chosen polymorphisms, demographic variables, and comorbidities, and FTND, HSI, CPD and TTF. Age was dichotomized with the use of a cut-off point of 45 years, since the median value of age in the sample was 44.2 years. FTND, HSI, CPD and TTF were incorporated into the analysis as dichotomous traits with FTND 4+, HSI 3+, CPD 20+ and TTF ≤ 5 min as cut-off points. These cut-off points were chosen for differentiating smokers with higher level of ND from those with lower level of ND. Because smoking quantity as a proxy phenotype for ND has been recognized to have the strongest association with SNPs at *CHRNA5*-*CHRNA3*-*CHRNB4* nicotinic receptor subunit gene cluster, the correlations between rs16969968, rs578776 and rs12914008 and CPD were additionally assessed.

The results were presented as odds ratios (ODs) with their 95% confidence intervals (95% CI). A significance level of 0.05 with was set for a type 1 error in all analyses. The Bonferroni correction procedure for multiple testing was used whenever appropriate.

## Results

The study sample consisted of 969 current smokers: 361 women aged 20–82 years (mean age 46.6 ± 11.9 years) and 608 males aged 19–85 years (mean age 42.7 ± 15.2 years). The mean CPD, FTND and HSI scores in the study sample were 17.3 ± 7.7, 3.9 ± 2.3 and 2.6 ± 1.5, respectively. Women smoked on average 14.6 ± 7.8 cigarettes per day, and men smoked 16.9 ± 8.9. One hundred and fourteen (11.8%) subjects met the definition for heavy smokers, and 108 (11%) for chippers. Among 285 subjects who self-reported any concomitant chronic diseases, the most prevalent were cardiovascular diseases, followed by diseases of respiratory system. There were 12 (1.2%) subjects with concomitant psychiatric disorders and 12 (1.2%) subjects with a history of neoplasm. The demographic data, comorbidities, and smoking profile of the study sample are shown in Table 1.

**Table 1:** **A smoking profile and comorbidities in the Kashubian sample of current smokers**

**Characteristics**	**Values: ** **N (%), ** **Mean ± ** **SD**
Males	608 (62.7)
Age (years)	44.2 ± 14.2
Age category:	
<30 yrs	194 (20.0)
30-45 yrs.	283 (29.2)
46-60 yrs.	384 (39.6)
>60 yrs.	108 (11.1)
Daily smokers	902 (93.1)
Tobacco chippers	107 (11.0)
Heavy smokers	114 (11.8)
Age at starting smoking (years)	19.1 ± 4.2
Smokers who started smoking in the age ≤16 yrs	206 (21.3)
Duration of smoking; years	23.0 ± 13.1
No. of cigarettes smoked daily	17.3 ± 7.7
FTND*	3.9 ± 2.3
HSI*	2.6 ± 1.5
TFC ≤5 min	230 (25.5)
Subjects with any chronic comorbidities	285 (29.4)
Respiratory diseases	67 (6.9)
Cardiovascular diseases	148 (15.3)
Gastrointestinal tract diseases	20 (2.1)
Degenerative spine and/or joint disease	40 (4.1)
Endocrine disease	59 (6.1)
Psychiatric disorders	12 (1.2)
Other	40 (4.1)
Subjects with a history of neoplasm	12 (1.2)

*FTND and HSI scores were 0 in the occasional smokers.

All subjects successfully underwent genotyping. The distributions of genotypes for rs16969968, rs578776, and rs12914008 did not deviate to any appreciable extent from expectations predicted by the Hardy-Weinberg equilibrium (p = 0.66, p = 0.68, and p = 0.66, respectively). The frequencies of alleles and genotypes for the three studied SNPs found in the Kashubian sample did not differ significantly from those downloaded from the HapMap CEU reference population (www.hapmap.org), except for the variant allele A of rs16969968. This variant was significantly less frequent in the Kashubian sample (p = 0.02). The frequencies of alleles of rs16969968 in *CHRNA5*, rs578776 in *CHRNA3*, and rs12914008 in *CHRNB4*, and the frequencies of genotypes found in the sample are provided in Table 2.

**Table 2:** **Frequencies of alleles and genotypes in rs16969968**-, **rs578776**-, **and rs12914008**-tagging loci in the studied sample and in the HapMap CEU* **reference population**

**Polymorphism**	**Allele/ ** **genotype**	**Frequency (%)**		***p*** **value**
		**Kashubians** **(North Poland)**	**HapMap CEU/** Global MAF**	
rs16969968 (*CHRNA5*)	A	30.6	38.5	**0.02**
	G	69.4	61.5	
	AA	9.7	15.9	**0.04**
	AG	41.8	45.1	0.50
	GG	48.5	38.9	**0.05**
rs578776 (*CHRNA3*)	C	72.1	75.2	0.33
	T	27.9	24.8	
	CC	51.7	56.6	0.32
	CT	40.9	37.2	0.45
	TT	7.4	6.2	0.63
rs12914008 (*CHRNB4*)	A	4.6	3.3/1.7	0.68
	G	95.4	96.7	
	AA	0.3	0	0.42
	AG	8.6	6.7	0.78
	GG	91.1	93.3	0.72

*HapMap Data Release 27 Phase II + III, February 2009.

**Global MAF in 1000 Genome Phase1 genotype data from 1094 worldwide individuals released in the May 2011 dataset.

Values in boldface denote a significant association.

In multivariate logistic regression analysis, there were no association between ND defined by FTND, HSI or TTF and age (Table 3). In turn, heavy smoking was significantly associated with older age (OR = 1.72, 95% CI: 1.14-2.59, p = 0.009), and men were more likely than women to be heavy smokers (OR = 1.70, 95% CI: 1.09-2.65, p = 0.018) (Table 3). Women were more likely than men to be physically non-dependent to nicotine, i.e., smoked up to 5 cigarettes daily or smoked occasionally (OR = 1.87, 95% CI: 1.25-2.82; p = 0.0025). However, having been a chipper was not associated with age (OR = 0.87, 95% CI: 0.58-1.31; p = 0.52).

**Table 3:** **Age**, **sex and chronic comorbidity associations with selected nicotine dependence measures**

**Variables**	**FTND No. of subjects**		**OR (** **95** **% CI) ** ***p*** **-value**	CPD* **No. of subjects**		**OR** ** (95% ** **CI) ** ***p-*** **value**	**HSI No. of subjects**		**OR (** **95% ** **CI)** ***p*** **-value**	**TTF No. of subjects**		**OR** **(95% ** **CI)** ***p*** **-value**
	**<4**	**≥4**		**≤20**	**>20**		**<3**	**≥3**		**≤5 min**	**>5 min**	
Females	170	191	1	293	31	1	151	173	1	100	261	1
Males	270	338	1.17 (0.90-1.53) 0.24	495	83	**1.70** (**1.09**-**2.65**) **0.018**	284	294	1.04 (0.80-1.37) 0.75	130	478	0.74 (0.55-1.01) 0.05
Age <45 years	233	244	1	396	44	1	217	223	1	102	375	1
Age ≥45 years	207	285	1.25 (0.95-1.64) 0.10	392	70	***1*** *.* ***72*** *(* ***1*** *.* ***14*** *-* ***2*** *.* ***59*** *)* ***0*** *.* ***009***	218	244	1.07 (0.80-1.42) 0.64	128	364	1.16 (0.84-1.60) 0.35
Chronic comorbidity -	324	360	1	560	77	1	363	321	1	152	532	1
Chronic comorbidity +	116	169	1.22 (0.91-1.65)0.18	228	37	1.00 (0.92-1.09) 0.97	139	146	1.17 (0.87-1.57) 0.31	78	207	1.22 (0.87-1.71) 0.24

*Only daily smokers.

Values in boldface denote a suggestive association and values in bold italics denote the significant association when applying Bonferroni correction for multiple testing (significance level: p = 0.0125).

ORs are adjusted for the two remaining variables.

Health status as an independent variable dichotomized into the presence and absence of any chronic disease did not significantly influence ND defined by FTND, CPD, HSI, and TTF (Table 3).

No association was also found between particular categories of concomitant chronic diseases, i.e., respiratory diseases, cardiovascular diseases, or a history of neoplasms with four studied phenotypes of ND (data not shown), except for psychiatric disorders that were significantly associated with the higher OR for TTF ≤5 min (OR = 4.16, 95% CI: 1.30-13.30, p = 0.016). The risk of smoking the first cigarette up to 5 minutes after wakening, after adjusting for age and psychiatric disorders, tended to be higher in women than in men (OR = 1.33; 95% CI: 0.98-1.80, p = 0.07).

The risk of starting smoking up to 16 years of age was significantly lower in women (OR after adjusting for age and psychiatric disorders = 0.53; 95% CI: 0.37-0.74, p = 0.0002).

Logistic regression results for the associations between ND defined by FTND, CPD, HSI or TTF and selected genotype variables are presented in Table 4. Risk allele at rs16969968 was found to be significantly associated with smoking more than 20 CPD. However, the *p*-value reached only a borderline significance after Bonferroni correction for the multiple testing.

**Table 4:** **Logistic regression results for the association between smoking more than 20 cigarettes per day and selected polymorphisms**

**SNP/** **Genotype**	**FTND No. of subjects**		**OR** **(95% ** **CI)** ***p*** **-value**	CPD* No. of subjects		**OR** ** (95% ** **CI) ** ***p*** **-value**	**HSI No. of subjects**		**OR** ** (95% ** **CI)** ***p*** **-value**	**TTF No. of subjects**		**OR** ** (95% ** **CI) ** ***p*** **-value**
	**<4**	**≥4**		**≤20**	**>20**		**<3**	**≥3**		**≤5 min**	**>5 min**	
rs16969968 at *CHRNA5*/												
GG	218	252	1	397	44	1	251	219	1	103	367	1
AA + AG	222	277	1.08 (0.83-1.40) 0.56	391	70	**1.63** (**1.09**-**2.45**) 0.017**	251	248	1.13 (0.88-1.46) 0.33	127	372	1.22 (0.91-1.65) 0.19
rs578776 at *CHRNA3*/												
CC	232	269	1	403	62	1	264	237	1	119	382	1
TT + TC	208	260	1.09 (0.84-1.41) 0.52	385	52	0.89 (0.60-1.30) 0.56	238	230	1.08 (0.84-1.39) 0.55	111	357	0.99 (0.73-1.33) 0.94
rs12914008 at *CHRNB4*/												
GG	398	485	1	717	105	1	455	428	1	209	674	1
AA + AG	42	44	0.86 (0.55-1.35) 0.52	71	9	0.87 (0.42-1.80) `0.70	47	39	0.88 (0.57-1.38) `0.58	21	65	1.04 (0.61-1.77) 0.90

*Only daily smokers.

**A rounded down *p*-value. The exact *p*-value is 0.01756.

The values in boldface denote the suggestive association when applying Bonferroni correction procedure for multiple testing (significance level: p = 0.017).

ORs are adjusted for age, sex and remaining polymorphisms.

## Discussion

In general, a low to moderate level of ND defined by FTND and HSI scores was found in the studied sample of Kashubians. In addition, in comparison to data obtained from the Global Adult Tobacco Survey (GATS) on tobacco use in 16 low-income and middle-income countries including Poland, Kashubians smoked on average fewer cigarettes daily (females and males, 14.6 and 16.9, respectively) than smokers in the general Polish population (females and males, 15.5 and 18.3, respectively) [36].

The risk of heavy smoking was significantly higher in smokers aged 45 years and older and males were more likely to be heavy smokers. Similar to other reports, women were more likely than men to be chippers, i.e., smokers physically non-dependent to nicotine. However, they were more likely, although not significantly, to report behaviour indicating physiological addiction (time to first cigarette) [37-39]. In concordance with reports from different countries and populations, they also started smoking later than men [39-41].

Our study showed that coexistence of chronic disease/diseases did not influence ND. Similarly, an analysis of the association between the coexistence of particular categories of comorbidities and ND did not show any significance, except for mental illnesses which are recognized as a risk for tobacco use and ND [42]. Even respiratory and cardiovascular system diseases that are commonly known to be related to or that are aggravated by cigarette smoking did not have any significant impact on ND. The only significant association that was found in subjects with psychiatric disorders related to an increased risk for TTF <5 min. Among those subjects, women were more likely than men to smoke their first cigarette within 5 min of wakening. In turn, we did not find an association between psychiatric disorders and CPD 20+, although there is evidence in the literature indicating this relationship [43-45]. Non-replication of the association could have been caused by several different factors. It has been suggested that heavy smoking and the urgency to smoke, which corresponds to TTF, may not share the same aetiology [46,47]; this may be one of the factors resulting in the non-replication.

The influence of *CHRNA5* rs16969968 on CPD has been firmly established [27,28]. The results of our study are in agreement with existing evidence that the *CHRNA5* rs16969968 polymorphism is associated with CPD as a proxy for ND. The OR for heavy smoking was 1.63 (95% CI: 1.09-2.45; p = 0.017) in carriers of the rare variant of this polymorphism, i.e., subjects with the AA or AG genotype, compared to non-carriers. However, after applying Bonferroni correction for multiple testing, this association appeared to be only borderline significant. The relatively small sample size was among the factors that may have hindered demonstrating a robust association. In addition, the lower minor allele frequency (MAF) of rs16969968 in the Kashubian population (30.6%) than that in other populations of Caucasian origin might have diminished the statistical power of our analysis. For instance, the MAF was 38.5% in the CEU population (HapMap Data Rel 27 Phase II + III, Feb. 2009) and 35.0% in the European-American sample analysed in the study by Saccone et al. [23]. If that was the case, the low MAF (4.6%) of another studied locus, *CHRNB4* rs12914008, which was confirmed in the Kashubian population, may have required a much larger population sample to find any association between that polymorphism and CPD. We decided to study this locus for two reasons. Firstly, the frequencies of minor and major alleles in *CHRNB4* rs12914008 in Kashubians had not been studied before. Secondly, this locus seemed intriguing because evidence of the involvement of this locus in ND had not yet been clearly established. Initially, *CHRNB4* rs12914008 was found to exert a protective effect against ND via its rare minor allele, with an estimated MAF of 4.5% [23]. However, another meta-analysis including 34 datasets from subjects of European ancestry did not show such effects of this locus on ND phenotypic status, defined by a dichotomous CPD measure [24]. The association between this variant and multi-item measures of ND has not been widely studied to date. To the best of our knowledge, only Sarginson et al. [25] tested the association between rs12914008 and the modified FTQ, demonstrating a negative result. Therefore, we included rs12914008 in our analysis of ND in Kashubians to investigate the potential role of this polymorphism in ND.

In spite of the evidence that the minor allele T of *CHRNA3* rs578776 is associated with a decreased CPD value, i.e., it confers protection against ND [23,24,48], we did not replicate such an association in the study group. Once again, this may be attributed to an insufficient sample size and a reduced statistical power.

In our study, four measures of ND were applied, including FTND, CPD, TTF, and HSI, to investigate which among them, if any, were most influenced by age, sex, comorbidities and the chosen polymorphisms. The FTND has been accepted as a standard measure in both clinical and research settings. This measure encompasses both physiological and psychological (cognitive and behavioural) aspects of dependence, although some evidence has suggested that it has poor psychometric properties and does not cover all important aspects of dependence, such as cravings, compulsion to smoke, nicotine withdrawal symptoms, behavioural saliency, and behavioural atomaticity, which are often regarded as the core construct of ND [49-52]. Other studies have demonstrated that the indices of psychological dependence could explain approximately 20% of the variance in FTND scores [53]. More recently, it was found that FTND is an instrument that primarily taps into behaviours that reflect how smokers cope with nicotine withdrawal [54]. The lack of association of FTND with studied demographic variables as well as chosen polymorphisms may result in part from these properties of FTND. Finding the association between FTND and a given polymorphism can be especially difficult, since a single SNP may influence a strictly defined aspect of physiological or psychological ND [55].

Therefore, in addition to FTND, we used two single FTND-derived items CPD and TTF alone as proxy measures of ND. We found that heavy smoking, which is a strong indicator of physiological ND, was significantly associated with age and marginally associated with sex and the AA + AG genotype at the CHRNA5 rs16969968 *locus*. The second item of HSI, TTF, was significantly associated only with psychiatric disorders. However, because of the small number of subjects with concomitant psychiatric disorders this association should be considered with caution.

Finally, we applied the HSI - a composite of CPD and TTF - as a measure of ND. Contrary to the FTND, the HSI emphasizes physiological dependence only, characterized by a smoker’s desire to maintain blood nicotine levels [19]. Since the average HSI score was 2.6 ± 1.5 in our sample, we applied a cut-off point of 3 to categorize smokers as having low/medium or high ND, as opposed to a cut-off point of 4, which was commonly used in the literature [56]. However, all studied variables in our analysis did not influence HSI.

There are several strengths of this study that should be highlighted. Firstly, we focused on a geographically and culturally isolated population so that the study sample was mostly genetically and culturally homogenous. Thus, we attempted to alleviate potential concerns regarding population stratification. Secondly, our analyses considered the possible influence of comorbidities on ND. We attempted to study which chronic concomitant disease modified ND traits. Thirdly, since our study group consisted of nonrelated subjects, we attempted to avoid those who could share common family environmental influences on ND.

The study also had limitations. Firstly, the study sample was not representative of the whole population of Kashubian smokers. In saying this, design of the study required this for convenience sampling. Therefore, non-related participants and current smokers were non-randomly recruited from patients and blood donors attending chosen medical centres or blood donations centres. Taking into account the considerable proportion of subjects with concomitant chronic diseases - many of which could influence the ND measures - the results of regression analyses were adjusted for the presence of comorbid conditions. Additionally, participants’ self-reports of their concomitant chronic diseases were not confirmed by a formal clinical interview or by checking medical documentation. This may have led to underreporting, especially in case of psychiatric disorders.

Secondly, only age and sex as basic demographic variables were included in the multivariate analysis, while factors known to be strongly correlated with smoking and ND, such as socioeconomic status or marital status, were not studied [57]. However, since we aimed to incorporate three SNPs at the *CHRNA5*-*CHRNA3*-*CHRNB4* subunit gene cluster as genetic factors in the regression analysis, incorporating more socioeconomic variables would have demanded a significantly larger sample to detect any relevant association after applying Bonferroni correction.

Nevertheless, our sample size and statistical power appeared to be too small to confirm statistical significance of the association between the *CHRNA5* rs16969968 polymorphism and CPD. Small sample size is considered one of the most common reasons for failure to replicate reported associations across studies [58]. Since the genetic background of ND probably requires the contribution of many genes of small effect, gene-gene interactions as well as gene-environment interactions should be evaluated to help explain the risk of developing a trait as complex as smoking. However, detecting these types of genetic effects and interactions requires samples of tens to hundreds of thousands of subjects. On the other hand, Bonferroni correction is considered a highly conservative method for multiple statistical comparisons [59] and was possibly too stringent for our attempts to replicate previously found robust associations between variants of the *CHRNA5*-*A3*-*B4* region and ND.

Another limitation of the study was the self-reporting of smoking quantity, which was not verified biochemically by the measurement of nicotine metabolites. Therefore, one can expect some degree of misreporting of smoking behaviour by smokers (e.g., reporting that they smoke fewer cigarettes than they actually do) [60]. Finally, both the Kashubian ancestry and the lack of first-degree relatives among participants of the study were based on self-reports, and were not verified at the genetic level. Therefore, we could not confirm the genetic homogeneity of the studied population or exclude individuals that were unexpectedly related to each other.

Despite its potential limitations, the present study is the only available estimate of ND in an ethnically and culturally homogenous population of Kashubians. We consider that isolated populations could serve as good samples for identifying genetic and environmental determinants of tobacco use and ND. Unfortunately, in current times of increasing cultural and genetic intermixing between populations, it may be difficult to find an ethnically and culturally homogenous sample in the future.

## Conclusions

To conclude, a low to moderate level of ND was observed among smokers in the sample of Kashubians. Age and sex have influence on CPD and it is likely that psychiatric comorbidities may influence TTF. Female sex is associated with a lower risk for starting smoking under the age of 16 years. In addition, the present study confirms that *CHRNA5* rs16969968 may contribute to the complex biological role of the *CHRNA5*-*A3*-*B4* gene cluster on the risk of ND, as defined by the number of cigarettes smoked daily. Efforts to better recognize ND are of great importance so that new approaches can be developed to reduce tobacco use, especially cigarette smoking. The continued identification of environmental factors and the search for genes involved in the development of ND will help in improving treatment measures for smoking cessation. Adjustment of treatments to personal environmental determinants and implementation of novel drugs tailored to personal genetic backgrounds and the stage of an individual’s ND may significantly increase the efficacy of treatment.

## References

[1] Eriksen M, Mackay J, Ross H (2011). Tobacco Atlas.

[2] Johnson EO, Chase GA, Breslau N (2002). Persistence of cigarette smoking: familial liability and the role of nicotine dependence. Addiction.

[3] Ho MK, Tyndale RF (2007). Overview of the pharmacogenomics of cigarette smoking. Pharmacogenomics J.

[4] Mayhew KP, Flay BR, Mott JA (2000). Stages in the development of adolescent smoking. Drug Alcohol Depend.

[5] True WR, Heath AC, Scherrer JF, Waterman B, Goldberg J, Lin N (1997). Genetic and environmental contributions to smoking. Addiction.

[6] Kendler KS, Thornton LM, Pederson NL (2001). Tobacco consumption in Swedish twins reared apart and reared together. Arch Gen Psychatry.

[7] Lessov CN, Martin NG, Statham DJ, Todorov AA, Slutske WS, Bucholz KK (2004). Defining nicotine dependence for genetic research: Evidence from Australian twins. Psychol Med.

[8] Lessov-Schlaggar CN, Pergadia ML, Khroyan TV, Swan GE (2008). Genetics of nicotine dependence and pharmacotherapy. Biochem Pharmacol.

[9] Munafό MR, Johnstone EC (2008). Genes and cigarette smoking. Addiction.

[10] Maes HH, Sullivan PF, Bulik CM, Neale MC, Prescott CA, Eaves LJ (2004). A twin study of genetic and environmental influences on tobacco initiation, regular tobacco use and nicotine dependence. Psychol Med.

[11] Li MD (2006). The genetics of nicotine dependence. Curr Psychiatry Rep.

[12] Bierut LJ, Madden PA, Breslau N, Johnson EO, Hatsukami D, Pomerleau OF (2007). Novel genes identified in a high-density genome wide association study for nicotine dependence. Hum Mol Genet.

[13] Saccone SF, Hinrichs AL, Saccone NL, Chase GA, Konvicka K, Madden PA (2007). Cholinergic nicotinic receptor genes implicated in a nicotine dependence association study targeting 348 candidate genes with 3713 SNPs. Hum Mol Genet.

[14] Stolerman IP, Jarvis MJ (1995). The scientific case that nicotine is addictive. Psychopharmacology (Berl).

[15] Pidoplichko VI, DeBiasi M, Williams JT, Dani JA (1997). Nicotine activated and desensitizes midbrain dopamine neurons. Nature.

[16] Salokangas RK, Vilkman H, Ilonen T, Taiminen T, Bergman J, Haaparanta M (2000). High levels of dopamine activity in the basal ganglia of cigarettes smokers. Am J Psychiatry.

[17] Koob GF (1992). Neural mechanisms of drug reinforcement. Ann NY Acad Sci.

[18] Heatherton TF, Kozlowski LT, Frecker RC, Fagerström KO (1991). The Fagerström test for nicotine dependence: a revision of the Fagerström tolerance questionnaire. Br J Addict.

[19] Heatherton TF, Kozlowski LT, Frecker RC, Rickert W, Robinson J (1989). Measuring the heaviness of smoking: using self-reported time to the first cigarette of the day and number of cigarettes smoked per day. Br J Addict.

[20] Bierut LJ, Stitzel JA, Wang JC, Hinrichs AL, Grucza RA, Xuei X (2008). Variants in nicotinic receptors and risk for nicotine dependence. Am J Psychiatry.

[21] Berrettini W, Yuan X, Tozzi F, Song K, Francks C, Chilcoat H (2008). Alpha-5/alpha-3 nicotinic receptor subunit alleles increase risk for heavy smoking. Mol Psychiatry.

[22] Weiss RB, Baker TB, Cannon DS, von Niederhausern A, Dunn DM, Matsunami N (2008). A candidate gene approach identifies the CHRNA5-A3-B4 region as a risk factor for age-dependent nicotine addiction. PLoS Genet.

[23] Saccone NL, Wang JC, Breslau N, Johnson EO, Hatsukami D, Saccone SF (2009). The CHRNA5-CHRNA3-CHRNB4 nicotinic receptor subunit gene cluster affects risk for nicotine dependence in African-Americans and European-Americans. Cancer Res.

[24] Saccone NL, Culverhouse RC, Schwantes-An TH, Cannon DA, Chen X, Cichon S (2010). Multiple independent loci at chromosome 15q25.1 affect smoking quantity: a meta analysis and comparison with lung cancer and COPD. PLoS Genet.

[25] Sarginson JE, Killen JD, Lazerroni LC, Fortman SP, Ryan HS, Schatzberg AF (2011). Markers in the 15q24 nicotine receptor subunit gene cluster (CHRNA5-A3-B4) predict severity of nicotine addiction and response to smoking cessation therapy. Am J Med Genet Part B Neuropsychiatr Genet.

[26] Caporaso N, Gu F, Chatterjee N, Sheng-Chih J, Yu K, Yeager M (2009). Genome-wide and candidate gene association study of cigarette smoking behaviors. PLoS One.

[27] Ware JJ, van den Bree MB, Munafò MR (2011). Association of the CHRNA5-A3-B4 gene cluster with heaviness of smoking: a meta-analysis. Nicotine Tob Res.

[28] Chen LS, Saccone NL, Culverhouse RC, Bracci PM, Chen CH, Dueker N (2012). Smoking and genetic risk variation across populations of European, Asian, and African American ancestry–a meta-analysis of chromosome 15q25. Genet Epidemiol.

[29] Broms U, Wedenoja J, Largeau MR, Korhonen T, Pitkäniemi J, Keskitalo-Vuokko K (2012). Analysis of detailed phenotype profiles reveals CHRNA5-CHRNA3-CHRNB4 gene cluster association with several nicotine dependence traits. Nicotine Tob Res.

[30] Pearce MS, Mann KD, Singh G, Davison B, Sayers SM (2014). Prevalence and validity of self-reported smoking in Indigenous and non-Indigenous young adults in the Australian Northern Territory. BMC Public Health.

[31] Unger JB (2014). Cultural influences on substance use among Hispanic adolescents and young adults: findings from Project RED. Child Dev Perspect.

[32] Wyniki Narodowego Spisu Powszechnego Ludności i Mieszkań, 2011. Podstawowe informacje o sytuacji demograficzno-społecznej ludności Polski oraz zasobów mieszkaniowych. [Results of the General Census and the Housing Stock Assessment, 2011. Basic information related to the socio-demographics of Polish population and the housing stock]. Central Statistical Office 2011. http://www.kaszubi.pl/images/dodatkowe/aktualnosci/Wyniki_demograficzne_spisu_2011_-_f.pdf

[33] Fagerstrom KO (1978). Measuring degree of physical dependence to tobacco smoking with reference to individualization of treatment. Addict Behav.

[34] Schiffman S (1989). Tobacco “chippers” – individual differences in tobacco dependence. Psychopharmacology (Berl).

[35] Barrett JC, Fry B, Maller J, Daly MJ (2005). Haploview: analysis and visualization of LD and haplotype maps. Bioinformatics.

[36] Giovino GA, Mirza SA, Samet JM, Gupta PC, Jarvis MJ, Bjala N (2012). Tobacco use in 3 billion individuals from 16 countries: an analysis of nationally representative cross-sectional household surveys. Lancet.

[37] Royce JM, Corbett K, Sorensen G, Ockene J (1997). Gender, social presuure, and smoking cessations: the Community Intervention Trial for Smoking Cessation (COMMIT) at baseline. Soc Sci Med.

[38] Champagne BM, Sebrié EM, Schargrodsky H, Pramparo P, Boissonnet C, Wilson E (2010). Tobacco smoking in seven Latin American cities: the CARMELA study. Tob Control.

[39] Hukkinen M, Kaprio J, Broms U, Koskenvuo M, Korhonen T (2009). Characteristics and consistency of light smoking: long-term follow-up among Finnish adults. Nicotine Tob Res.

[40] Filippidis FT, Vardavas CI, Loukopoulou A, Behrakis P, Connolly GN, Tountas Y (2013). Prevalence and determinants of tobacco use among adults in Greece: 4 year trends. Eur J Public Health.

[41] Memon A, Moody PM, Sugathan TN, el-Gerges N, Al-Bustan M, al-Shatti A (2000). Epidemiology of smoking among Kuwaiti adults: prevalence, characteristics, and attitudes. Bull World Health Organ.

[42] Ziedonis D, Hitsman B, Beckham JC, Zvolensky M, Adler LE, Audrain-McGovern J (2008). Tobacco use and cessation in psychiatric disorders: National Institute of Mental Health report. Nicotine Tob Res.

[43] John U, Meyer C, Rumpf HJ, Hapke U (2004). Smoking, nicotine dependence and psychiatric comorbidity–a population-based study including smoking cessation after three years. Drug Alcohol Depend.

[44] Wehring HJ, Liu F, McMahon RP, Mackowick KM, Love RC, Dixon L (2012). Clinical characteristics of heavy and non-heavy smokers with schizophrenia. Schizophr Res.

[45] Haustein KO, Haffner S, Woodcock BG (2002). A review of the pharmacological and psychopharmacological aspects of smoking and smoking cessation in psychiatric patients. Int J Clin Pharmacol Ther.

[46] Richardson CG, Ratner PA (2005). A confirmatory factor analysis of the Fagerström Test for Nicotine Dependence (FTND). Addict Behav.

[47] Marks MJ, Pauly JR, Gross SD, Deneris ES, Hermans-Borgmeyer I, Heinemann SF (1992). Nicotine binding and nicotinic receptor subunit RNA after chronic nicotine treatment. J Neurosci.

[48] Conlon MS, Bewick MA (2011). Single nucleotide polymorphisms in CHRNA5 rs16969968, CHRNA3 rs578776, and LOC123688 rs8034191 are associated with heaviness of smoking in women in Northeastern Ontario, Canada. Nicot Tob Res.

[49] Moolchan ET, Radzius A, Epstein DH, Uhl G, Gorelic DA, Cadet JL (2002). The Fagerstrom test for nicotine dependence and the diagnostic interview schedule: do they diagnose the same smokers?. Addict Bahav.

[50] Shadel WG, Shiffman S, Niaura R, Nichter M, Abrams DB (2000). Current models of nicotine dependence: what is known and what is needed to advance understanding of tobacco etiology among youth. Drug Alcohol Dependence.

[51] Etter JF, Duc TV, Perneger TV (1999). Validity of the Fagerström test for nicotine dependence and of the Heaviness of Smoking Index among relatively light smokers. Addiction.

[52] Etter JF (2005). A comparison of the content-, construct- and predictive validity of the cigarette dependence scale and the Fagerström test for nicotine dependence. Drug Alcohol Depend.

[53] Dijkstra A, Tromp D (2002). Is the FTND a measure of physical as well psychological tobacco dependence?. J Subst Abuse Treat.

[54] DiFranza JR, Wellman RJ, Savageau JA, Beccia A, Ursprung WW, McMillen R. What aspect of dependence does the Fagerström Test for Nicotine Dependence measure? Hindawi Publishing Corporation, ISRN Addiction, 2013:Article ID 906276, http://dx.doi.org/10.1155/2013/906276 10.1155/2013/906276 PMC4403618 25969829

[55] Lerman C, Swan GE (2002). Non-replication of genetic association studies: is DAT all, folks?. Nicot Tob Res.

[56] Chaiton MO, Cohen JE, McDonald PW, Bondy SJ (2007). The Heaviness of Smoking Index as a predictor of smoking cessation in Canada. Addict Behav.

[57] Pennanen M, Broms U, Korhonen T, Haukkala A, Partonen T, Tuulio-Henriksson A (2014). Smoking, nicotine dependence and nicotine intake by socio-economic status and marital status. Addict Behav.

[58] Cardon LR, Bell JI (2001). Association study designs for complex diseases. Nat Rev Genet.

[59] Li MD, Yoon D, Lee J-Y, Han B-G, Niu T, Payne TJ (2010). Associations of variants in CHRNA5/A3/B4 gene cluster with smoking behaviors in a Korean population. PLoS One.

[60] Patrick DL, Cheadle A, Thompson DC, Diehr P, Koepsell T, Kinne S (1994). The validity of self-reported smoking: a review and meta-analysis. Am J Public Health.

